# Exercise-mediated cerebrovascular repair in Alzheimer's disease: from pathophysiology to therapeutic precision

**DOI:** 10.3389/fnagi.2025.1632365

**Published:** 2025-09-16

**Authors:** Ming Cai, Keren Cai, Ziqi Wei, Jing Zhou, Jiayi Shu, Weiyi Wang, Wanju Sun, Jingyun Hu

**Affiliations:** ^1^College of Rehabilitation Sciences, Shanghai University of Medicine and Health Sciences, Shanghai, China; ^2^Central Lab, Shanghai Key Laboratory of Pathogenic Fungi Medical Testing, Shanghai Pudong New Area People's Hospital, Shanghai, China

**Keywords:** exercise, Alzheimer's disease, cerebral vessels, vascular function related cells, vascular factors

## Abstract

Cerebrovascular dysfunctions, encompassing changes in cerebrovascular microstructure, blood-brain barrier (BBB) integrity, cerebrovascular reactivity, and cerebral blood flow (CBF), accelerate the pathological progression of Alzheimer's disease (AD). Exercise emerges as a promising non-pharmacological intervention that enhances cerebrovascular repair for the treatment of AD. This review summarizes the pathological vascular changes in AD pathology, such as pericyte loss, endothelial dysfunction, and capillary fibrosis, which exacerbate hypoperfusion, hypoxia, and amyloidogenesis. We further discuss the contributing vascular factors and underlying signaling mechanisms to explore potential targets for AD diagnosis and therapy. Finally, we present evidence concerning the impact of exercise on cerebral vascular signaling and the cells involved in vascular plasticity. We also address the impact of various exercise patterns on cerebrovascular health. This work aims to uncover the potential and intervention effects of exercise on cerebrovascular non-malignant alterations and will provide exercise strategies for treating AD.

## 1 Introduction

Alzheimer's disease (AD) is a progressive neurodegenerative disease with the classical pathological features of plaques and neurofibrillary tangles, which accounts for accounts for 60%−80% of dementia cases (Li S. et al., 2021). The major clinical manifestations are brain structure changes and memory loss. Besides aging, physical inactivity is also one of the risk factors in AD, which reduces cerebral blood flow (CBF) and impairs cerebrovascular function (Livingston et al., 2020). Clinically, the drug development process targeting β-amyloid (Aβ) deposition and Tau protein hyperphosphorylation has a failure rate as high as 99.6%, with developed drugs unable to fundamentally cure the disease's symptoms. Moreover, treatment rate is below 21.1% (Jia et al., 2020), posing significant healthcare challenges for both developed and developing countries amidst the growing aging population.

Aβ accumulation within vascular smooth muscle cells has been proven to result in inflammatory responses, oxidative stress, compromised vasorelaxation, and impaired integrity of the blood-brain barrier (BBB; Stukas et al., 2014; Park et al., 2014; Kalheim et al., 2017). Tau pathology exacerbates vascular abnormalities and cellular senescence (Bryant et al., 2020, 2021). Moreover, the pathological development of AD has been proved to be related to the decrease in vascular density and CBF (Gallego et al., 2022; Bersini et al., 2020). In turn, capillary injury and blood flow shutdown trigger capillary loss and string vessel formation (Brown et al., 2009). Clinic research has demonstrated that 83% of AD patients suffered a mild degree of amyloid angiopathy, with ≥25.6% experiencing moderate to severe forms (Ellis et al., 1996). Therefore, the treatment of cerebral microangiogenesis will contribute to restoring neurovascular function and AD-related cognitive dysfunction (Zhang W. et al., 2018; Tong et al., 2009).

Exercise induces brain physiological changes at anatomic, cellular, and molecular levels by initiating a cascade of cellular and molecular processes (De la Rosa et al., 2020). It is well-established that physical activity substantially lowers the risk of AD (De la Rosa et al., 2020; Ren and Xiao, 2023; López-Ortiz et al., 2021) by inhibiting Aβ and releasing neurotrophins for brain health (De la Rosa et al., 2020; Valenzuela et al., 2020). Neuroimaging studies have shown that regular exercise enhances activity in the dorsolateral prefrontal cortex and limbic system, thereby improving functions such as emotion regulation, attention, executive function, and cognition (Zhou, 2020). Moreover, exercise is conducive to cerebral circulation and plasticity in AD (Li et al., 2023; Cass, 2017; Bliss et al., 2021), making it an effective strategy for addressing cerebrovascular dysfunction in AD pathology.

Despite compelling evidence supporting the neuroprotective effect of exercise in AD, critical knowledge gaps persist in translating these findings into clinically actionable strategies. This translation challenge requires understanding the mechanisms underlying the benefits of diverse exercise modalities. Notably, traditional Chinese exercises such as Tai Chi, as well as specific disciplines like equestrian skills and swordsmanship, are well-documented to have numerous beneficial effects, including neuroprotective, anti-inflammatory, anti-oxidative, nutritional, and longevity-promoting functions (Zhou, 2019). The shared and distinct cerebrovascular mechanisms through which such varied activities exert their positive effects, particularly on AD-related pathways, remain incompletely understood.

Given established benefits of exercise on cerebrovascular function and plasticity, investigating exercise-induced cerebrovascular remodeling may help bridge the critical gap in clinical translation by elucidating the mechanisms through which exercise influences brain metabolism in AD.

In this review, we summarize the current understanding of the abnormal cerebrovascular structures and metabolic changes in associated subcellular components in AD. We also discuss alterations in vascular factors and signaling pathways. We outline the effects of exercise on vascular function in AD and recent advances in the role of various exercise prescriptions on cerebrovascular health. Overall, our review highlights the necessity for further research to explore the potential of exercise as a therapeutic strategy for addressing cerebrovascular dysfunctions in AD.

## 2 AD and cerebral vessels

Cerebrovascular insufficiency, such as reduced blood supply to the brain or disrupted cerebrovascular integrity, may occupy an initiating or intermediate position in the pathological development of AD (Cacabelos et al., 2003; Farkas and Luiten, 2001). Cortical hypoperfusion occurs early in AD (Niwa et al., 2000, 2002), accompanied by vascular degeneration and reduced microvascular density (Brown and Thore, 2011). Morphological assessments have revealed microvascular degeneration in early-stage AD brains, characterized by decreased microvascular density, linear vessel atrophy, significant diameter reduction, and other microvascular alterations, all of which contribute to decreased CBF (Lau et al., 2020). Tau oligomers accumulate in AD cerebral microvessels, associating with endothelial cells and compromising microvascular integrity (Castillo-Carranza et al., 2017).

Furthermore, cerebral vascular degeneration and reduced blood flow lower the efficiency of Aβ clearance. Aβ deposition in the cerebral vascular system causes decreased brain perfusion, promotes ischemic damage, while also triggering vascular and neuronal degeneration that ultimately impairs cognition (Di Marco et al., 2015). Despite this, AD cerebral microvasculature releases vascular endothelial growth factor (VEGF), interleukin-1β (IL-1β), IL-6, IL-8, angiogenic factors, HIF-1α, and other angiogenesis-promoting factors to compensate. However, functional blood vessels do not increase. Notably, endogenous VEGF directly binds to Aβ peptides, leading to a decrease in VEGF activity, which further results in cerebral vascular degeneration and diminished neuroprotective effect (Patel et al., 2010). Therefore, there is a strong link between cerebral microvascular dysfunction and the onset of AD. A prospective study has demonstrated that managing cerebral vascular risk factors in AD patients can decelerate cognitive decline (Morrone et al., 2020), implying that enhancing the integrity of cerebral microvascular structure and function could offer a promising therapeutic avenue for managing AD clinically as a biomarker and therapeutic target for early diagnosis (Tregub et al., 2022).

### 2.1 Cerebral microvascular structure degeneration in AD

Cerebral microvessels, with a diameter ranging from 10 to 20 μm (Erdener and Dalkara, 2019), consist of peripheral cells, endothelial cells, basal membrane, and astrocytes (Krueger and Bechmann, 2010), forming an essential ultrastructure crucial for maintaining the BBB (Farkas and Luiten, 2001; Zlokovic, 2011). This vascular network includes micro arteries, micro veins, and capillaries, with the latter representing the finest branches that interconnect within a three-dimensional network (Farkas and Luiten, 2001). Recent studies highlight the critical role of microvessels for CBF control (Davis and Attwell, 2023; Zerbi et al., 2013) and energy fuel (Koepsell, 2020). Hence, the structural degeneration of cerebrovascular microstructure precedes both CBF (Farkas et al., 2000a) and cognitive impairment (Kelly et al., 2017) in the onset of AD.

**Figure 1:**
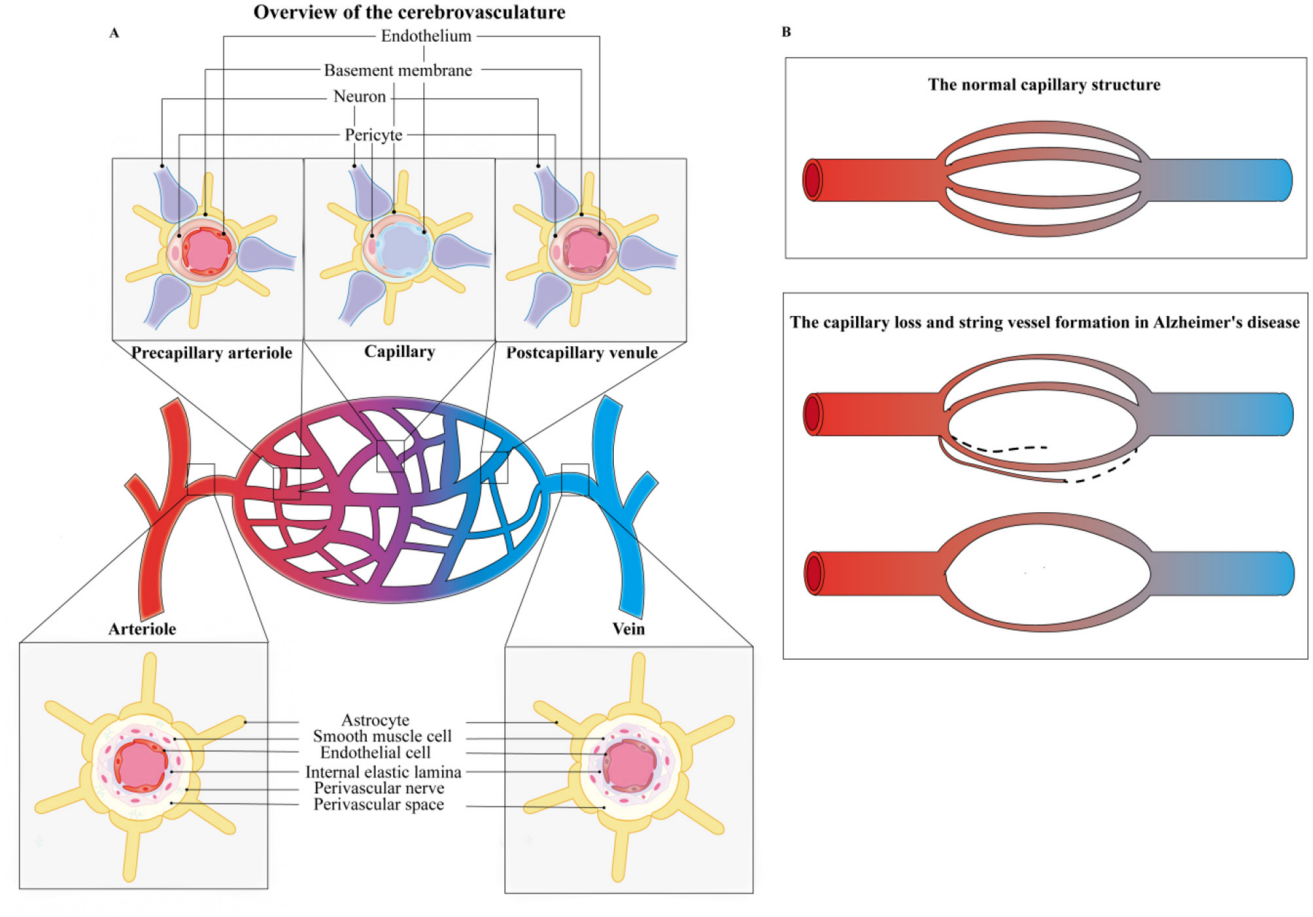
The changes in cerebral vessels in AD. **(A)** Overview of the cerebrovascular system. The schematic illustrates cross-sectional views of the cerebral microvasculature, including the arteriole, vein, precapillary arteriole, capillary, and postcapillary venule. The cell types of arterioles and veins primarily include astrocytes, smooth muscle cells, endothelial cells, and perivascular nerves. The cell types of precapillary arteriole, capillary, and postcapillary venule mainly contain endothelium, neuron, and pericyte. **(B)** Schematic of capillary structure. Compared to the normal cerebral vessels, the capillaries lose and string vessels shapes in AD.

Capillary fibrosis involves the thickening of the capillary basement membrane and the accumulation of collagen type IV, impairing nutrient transport and the clearance of waste products (Farkas et al., 2000a; Kalaria, 1996). Observations have shown that the ultrastructure of cerebral capillaries is detrimentally affected in AD (Kelly et al., 2017; Farkas et al., 2000b; Tomoto et al., 2023a), evidenced by a higher incidence of basement membrane thickening and collagen accumulation compared to age-matched individuals (Farkas et al., 2000b; Mancardi et al., 1980). These alterations manifest as irregularities in the abluminal surface, constriction, and dilatation patterns along the capillary paths (Kimura et al., 1991). Meanwhile, the terminal arterioles frequently had focal constriction and smooth muscle cells with an irregular shape and arrangement in the AD brain (Kimura et al., 1991). In the hippocampus, acoustic cortex, visual cortex, and parietal cortex, the capillaries exhibit numerous fusiform dilatations, tortuosities, abnormal branching, and fusion along with mitochondrial abnormalities in the endothelial cells, pericyte degeneration, and perivascular microglial proliferation (Baloyannis and Baloyannis, 2012). Notably, the observation of numerous string vessels, composed solely of collagen tubes without endothelial cells or a lumen, is indicative of severe capillary damage (Brown and Thore, 2011; Challa et al., 2004; Kolinko et al., 2021; Gama Sosa et al., 2010; Figure 1).

In 3 × Tg AD mice, vascular surface area declines as early as 3 months affecting regions like the hippocampal CA1, CA3, and DG subregions, cingulate cortex, and somatosensory cortex, preceding the formation of Aβ-plaques (Quintana et al., 2021). It reflects an early onset of microvascular degeneration before the formation of Aβ-plaques. Similarly, in APP/PS1 models, early microangiopathies are observed in mice aged 4–5 months, with conspicuous microaneurysms and protrusions in capillaries compared to wild-type counterparts (Kelly et al., 2017). These microvascular changes in AD mice progress further, with events of intracerebral microvascular leakage occurring by 7 months (Kelly et al., 2015), and alterations in vascular diameter, volume, and branch characteristics becoming evident by 24 months (Zhang et al., 2019).

The abnormal structure of cerebral capillaries overwhelmingly results in the breakdown of BBB (Winkler et al., 2014) at the expense of ineffective utilization of oxygen (O_2_) and glucose (Farkas et al., 2000a; Mooradian et al., 1997; Zenaro et al., 2017). For example, compared to age-matched peers, defective expression of glucose transport 1 (GLUT1) is observed in the cerebral microvessels of patients with AD (Harik, 1992; Horwood and Davies, 1994) and rodent models (Do et al., 2014), suggesting a lower glucose availability and uptake. Previous study has shown that glucose metabolism in the precuenus, posterior cingulate, and lateral parietal lobes was detected reduced earlier than 10 years before predicted AD using the [(18)F]flurodeoxyglucose PET (Benzinger et al., 2013). A similar recession pattern of glucose hypometabolism was also demonstrated in the late-middle-aged subjects with apolipoprotein E (APOE) epsilon4 compared to the non-carrying peers (Langbaum et al., 2010). This implies the possible early microcirculatory impairment in these brain regions. The dysfunctional endothelial cells or pericytes and morphological irregularities further trigger the excessive heterogeneity in capillary transport, which compromises the availability of O_2_ in tissues (Erdener and Dalkara, 2019). Subsequently, the deprivation of O_2_ and glucose further up-regulates the hypoxia inducible factor-1α (HIF-1α)-mediated β-secretase 1 (BACE1) for the expression of amyloid protein precursor (AβPP) that accelerates the Aβ_1 − 42_ production in brain capillary endothelial cells (Bulbarelli et al., 2012).

Apart from the above hypoxia and hypometabolism, pericyte detachment not only causes focal microhemorrhages but also the extravasation of serum proteins and red blood cells (RBCs; Zlokovic, 2011). Then, the toxic iron stored in the hemoglobin (Spencer and Mast, 2022) from RBCs catalyzes the reactive oxygen species (ROS) reaction and triggers oxidative stress (Dixon and Stockwell, 2014), in turn, neuronal damage (Zlokovic, 2011). Capillary pathology is also considered as the etiology for higher cerebrovascular resistance (CVR; Tomoto et al., 2023a). Yew et al. reported that the CVR index (mean arterial pressure/regional CBF) was elevated in AD patients due to capillary narrowing accelerating the microcirculation embolism (Murata et al., 2023). A 2-year follow-up revealed that the symptom of cognitive decline and brain atrophy in older individuals with higher CVR index were more pronounced, hinting at a risk of dementia (Yew et al., 2017).

### 2.2 Reduced cerebral blood flow

In addition to chemical changes, such as those involving CO_2_, the rate of CBF is also controlled by brain activity. As the activity of a brain region increases, the CBF in this region also grows to ensure adequate oxygen and nutrient delivery to active brain regions, a phenomenon known as “functional hyperaemia” (Zlokovic, 2011; Iadecola, 2004; Venkat et al., 2016). Brain function would immediately cease, leading to irreversible damage if CBF is interrupted (Iadecola, 2004). Therefore, disruption of CBF regulation, baseline, temporal, or regional homeostasis, may represent a major factor in the development and progression of AD (Claassen et al., 2021; Wierenga et al., 2014; Jeong et al., 2023). During the aging process, CBF typically decreases at a rate of 0.5% per year. In old age, there is already a 20% reduction in CBF, leading to an inadequate supply of nutrients to the brain. This insufficiency contributes to the exacerbation of neurodegenerative diseases (Zou et al., 2009; Zhou, 2017). Although abnormal vascular conditions and hypercapnia initially stimulate compensatory mechanisms (Zhou, 2017; Miller et al., 2018) to regulate CBF and increase the cerebrovascular reactivity (the ability of cerebral vessels to dilate or constrict in response to metabolic or chemical stimuli) for maintaining brain function, eventually blood flow decreases below the normal physiological range (Walek et al., 2023; Farkas et al., 2000c; van Beek et al., 2012). As a result, approximately 40% CBF in the brain regions of precuneus, hippocampus, posterior cingulate gyrus, temporal lobe, occipital lobe, and parietal lobe was reduced in AD patients compared to the peer elderly (Binnewijzend et al., 2013; Johnson et al., 2005; Du et al., 2006).

Low baseline perfusion correlates with accelerated cognitive decline (Wolters et al., 2017), inducing white matter lesions and cortical microinfarcts (Suter et al., 2002). Chronic hypoperfusion promotes capillary wall malformations, underlying mild memory deficits (Farkas et al., 2000a). Decreased CBF results in a chronically reduced supply of glucose and oxygen for the brain of neuronal metabolism (Horwood and Davies, 1994; Bell et al., 2010; Riederer et al., 2018; Love and Miners, 2016), especially in the posterior brain regions, which is correlated with declined global cognition and executive function in AD patients (Benedictus et al., 2017; Leeuwis et al., 2017). A multicenter study has manifested that a higher level of Aβ accumulation was highly associated with low CBF in the early stage of diagnostic AD (Mattsson et al., 2014). Although a single, mild, cerebral hypoperfusion seems to have little detrimental effect on both Tau and Aβ (Koike et al., 2010), a chronic hypoperfusion event is prone to the accumulation of Aβ in the hippocampus, especially the position of axonal terminals, which impairs the synaptic density and ultrastructure, causes a memory loss (Wang et al., 2010), and induces white matter lesions correlating with dementia (Brown and Thore, 2011; Zhou et al., 2015). Hence, CBF may be an early biomarker and a predictor of AD progression.

(Binnewijzend et al. 2013) reported that the uncorrected CBF of patients with AD was reduced to 27 ± 5 mL/100 g/min, especially in the regions of parietal lobes at the value of 22 ± 6 mL/100 g/min by using a whole-brain three-dimensional (3D) pseudocontinuous arterial spin-labeling (ASL) technique. Compared to the peer individuals, decreased cerebral blood supply is also reported to be lower by about 70% in the regions of the hippocampus and temporal cortex in AD patients (Farkas et al., 2000a; Eberling et al., 1992; Ohnishi et al., 1995). Furthermore, patients suffering from AD invariably are observed to reduce CBF in the regions of the hippocampus, inferior temporal cortex, precuneus, entorhinal cortex, and inferior parietal cortex (Mattsson et al., 2014; Ohnishi et al., 1995; DeKosky et al., 1990; O'Brien et al., 1992), with decreased velocity and increased pulsatility and resistance indices (Cacabelos et al., 2003) via the technology of neuroimaging modalities. Generally, the area of hypoperfusion is primarily in the inferior parietal cortex extending into the bilateral posterior cingulate gyri, bilateral superior, and middle frontal gyri, which is considered to be a pattern of topographical progression according to the dynamic pathologic processes from mild cognitive impairment (MCI) to AD (Johnson et al., 2005). It starts at the posterior cingulate gyrus with extension to the medial precuneus, then into the inferior parietal, lateral frontal, superior temporal, and orbitofrontal cortices (Dai et al., 2009).

Of notably, higher CVR (Tomoto et al., 2023a) and autoregulatory function (de Heus et al., 2018) seem not to be the chief culprits of CBF reduction in AD. Under these circumstances, cerebral microvessel dysfunction may play a prominent role in reduced CBF (Claassen et al., 2021; Kisler et al., 2017a). (Hamel et al. 2008) demonstrated cortical microvessel denervation and basal forebrain cholinergic terminal loss in AD, impairing acetylcholine synthesis. Supported evidence is that the vesicular acetylcholine transporter decreased in the cortex of patients with AD, implying an impaired function of arteriolar vasodilation and further reduced CBF (Hunter et al., 2012).

Degeneration of pericytes is believed as a critical factor for hypoperfusion at the aspect of vascular anatomical microstructure (Winkler et al., 2014; Love and Miners, 2016). (Bell et al. 2010) demonstrated that pericyte loss would contribute to the brain microcirculation reduction and, as a result, diminished capillary perfusion, CBF, and CBF responses to brain activation. Besides, the generation of Aβ produces reactive oxygen species (ROS) to release the endothelin-1 (ET-1), which evokes the ETA receptors at pericytes to constrict the capillaries (Nortley et al., 2019). Side effects of Aβ and ROS also impair the NO synthesis for vasodilatation and thus in turn CBF reduction (Toda et al., 2009a,b).

### 2.3 Loss of cerebral vessels

Vascular loss occurs in multiple brain regions in AD microvascular pathology. (Fischer et al. 1990) reported that the vascular density index of the prefrontal cortex, basal forebrain, sensorimotor cortex, and hippocampus decreased by 19.2%, 44.1%, 11.2%, and 30%, respectively, in postmortem patients with AD. Another study by Bell and Ball demonstrated a 17.9% reduction of capillary density in the postmortem calcarine cortex in AD patients (Bell and Ball, 1990). (Kitaguchi et al. 2007) found that the cortical microvessel density in the autopsied brains with AD was reduced, especially in close proximity to the location of plaques, implying a role of Aβ in cerebrovascular disease. A shred of direct evidence is the inhibitory effect of Aβ on the formation of human brain capillaries depending on the dosage, and moreover, a high dose of Aβ stimulates capillary degeneration *in vitro* study (Paris et al., 2004b). (Brown et al. 2007) revealed that vascular density decreased in the deep white matter of postmortal patients with AD depending on the age. (Baloyannis and Baloyannis, 2012) documented fewer capillaries in AD patients compared to healthy individuals, along with reduced tight junction length and number per vessel length in certain brain regions. Similarly, (Buée et al. 1994) found decreased microvascular density and abnormal vascular changes in specific layers of the frontal and temporal cortex, accompanied by neuron loss in these regions. In the severely affected cortical regions of the frontal and parieto-occipital cortex, the capillary network disappeared and capillaries collapsed via visualization by the silver impregnation technique (Suter et al., 2002).

Similar vascular changes have been observed in AD rodent models. (Lee et al. 2005) reported that the number of capillary segments in white matter was approximately 21% lower in the 7-month AAP/PS1 mice compared to the age-matched wild-type mice. (Paris et al. 2004a) reported that vascular densities began to descend in both the cortex and hippocampus at 3-month old in the APP mice, and more importantly, approximately a 30% reduction at 17-month old compared to the littermate control. (Quintana et al. 2021) demonstrated that vascular intervessel distance progressively increased in the hippocampal regions of CA1 and CA3 and cingulate cortex at the 3-month age and the total volume of capillary segments substantially dropped in the whole brain at the 24-month age of the 3 × Tg AD mice, suggesting a deficit of vessel density. (Alata et al. 2015) found that the cerebral vascular vessels were reduced by 26%−38% and the basement membranes thinned by 30%−35% in the 12-month-old APOE4 mice, reflecting evidence of vascular atrophy. Kolinko et al. found that the total capillary density of the hippocampus in the 12-month-old male APP/PS1 rats was lower than the normal littermates. However, surprisingly, there is a wide difference in the hippocampal subfields. For instance, although the number density in CA1 and DG regions was low, the capillary density of CA2 and CA3 was high (Kolinko et al., 2021), suggesting the changes in vascular density in AD are possibly different in different brain regions and subregions. Contradictorily, although the vascular length and microvessel area have reduced in AD patients or models (Gama Sosa et al., 2010), several research report that no significant changes or even increase in vascular density associated with multiple pathological factors like cerebral parenchymal tissue atrophy (Kolinko et al., 2021; Hunter et al., 2012; Fisher et al., 2022; Kirabali et al., 2020; Salat et al., 2001), special brain region associated with the age or disease progression [Fisher et al., 2022; Bell and Ball, 1981; e.g., hippocampus (Desai et al., 2009) or cerebral cortex (Giuliani et al., 2019; Xu et al., 2020)], genetic inheritance (Kolinko et al., 2021), early vascular compensatory increase or in formation of newer compensatory microvessel loops to response the cerebral hypoperfusion (Challa et al., 2004; Desai et al., 2009; Burke et al., 2014). The characteristics of these capillaries are narrowed and string, accompanied by a loss of vascular supply function (Challa et al., 2004; Hunter et al., 2012), predominantly occurring in severe neuronal lesion zones (Bell and Ball, 1981; Figure 1). Detection of vessel size and structure by neuroimaging technique may provide hallmarks for the pathological vessels of AD, and for monitoring the effects of vascular-targeted therapeutics.

## 3 Changes of cerebral vascular function related cells in AD brain

### 3.1 Mural cell death

#### 3.1.1 Perithelial cells

Pericytes were first described by (Rouget 1910) as a population of contractile cells surrounding capillaries. They are a type of vascular mural cells embedded within the basement membrane of blood microvessels (Kisler et al., 2017b; Armulik et al., 2011; Zlokovic, 2008) and serve as a critical component of the BBB (Armulik et al., 2010), covering up to 80% of brain capillaries in the human cortex and hippocampus (Sengillo et al., 2013). Pericytes play a vital role in regulating various neurovascular functions, including cerebral blood flow responses to neuronal activation, angiogenesis, neovascularization, maturation and maintenance of the BBB, vascular O_2_ supply for brain metabolism, regulation of capillary blood flow, and clearance of toxic byproducts from capillaries (Krueger and Bechmann, 2010; Bell et al., 2010; Kisler et al., 2017b; Daneman et al., 2010; Winkler et al., 2011).

The loss of pericytes in the cortex has been reported in the AD rodent model (Janota et al., 2015). I Postmortem AD brains show 35%−45% pericyte reduction in the frontal lobe (Ding et al., 2020). Sengillo et al. proved that the pericyte number decreased by 59% in the human AD cortex and 60% in the hippocampus, respectively. Subsequently, they declared that the pericyte coverage of capillary microvessels was reduced by 29%−32% in the cortex and 30%−33% in the hippocampus, marked by two labels of vascular mural cells-platelet-derived growth factor receptor beta (PDGFRβ) and CD13 (Sengillo et al., 2013). The work by Li et al. demonstrated that the absolute number of pericytes was decreased by 34% in the post-mortem AD patients (Kolinko et al., 2021), paralleled in the hippocampus of 6-month age 5 × FAD mice (Li P. et al., 2022). Several lines of evidence have proved that deficiency of brain pericytes led to BBB permeability (Armulik et al., 2010), vascular damage (Bell et al., 2010; Winkler et al., 2010), Tau protein hyperphosphorylation (Sagare et al., 2013), Aβ deposition (Sengillo et al., 2013), and white matter functional deficits (Montagne et al., 2018), which inevitably accelerated the process of AD. In the AD precuneus, the loss of PDGFRβ is positively correlated with the fibrinogen leakage, reduced oxygenation supply, and fibrillar Aβ accumulation (Miners et al., 2018). Interestingly, pericyte loss prompts the mice to develop chronic vascular damage preceding neuronal dysfunction (Winkler et al., 2010). Deficiency of PDGFRβ in the brain of mice contributes to capillary diminishment, CBF reduction, BBB disruption, and fibrin accumulation in the region of the cortex and hippocampus with age, resulting in the event of neuroplasticity impairment and neurodegeneration (Bell et al., 2010). In the PDGFRβ knockout APP mice, the mice exhibit an accelerated age-dependent loss of pericytes at 1 month of age and reach a 55% loss at 9 months. The levels of Aβ_1 − 40_ and Aβ_1 − 42_ in the cortex and hippocampus are far more than those of normal littermates and humans, even the APP littermates. In addition, deficient PDGFRβ accelerated the Tau pathology. These trigger neuron loss and vascular damage like BBB breakdown and cerebral microvessel reduction, which ultimately is conducive to the AD-like pathological phenotypes (Sagare et al., 2013). In turn, BBB dysfunction and diminished brain microcirculation give rise to vascular damage, which is caused by the loss of pericytes (Winkler et al., 2011). PDGFRβ knockout mice accumulate plasma-derived immunoglobulin G (IgG) at 1 month, leading to deposition of serum proteins and several macromolecules of vasotoxicity and neurotoxicity, and exhibited a higher degree of hypoxic situation associated with the chronic hypoperfusion in the hippocampus and cortex regions at the age of 6–8 months (Bell et al., 2010).

Aβ accumulation critically drives pathological factor of pericyte cell loss in progressive AD development. In patients with AD, fibrillar Aβ accumulation accelerates the PDGFRβ loss and pericyte degeneration in the precuneus (Miners et al., 2018). Vitro study has shown that various Aβ peptide treatments, such as HCHWA-D-Aβ_1 − 40_, D-Aβ_1 − 40_, or Aβ_1 − 42_, all would decrease the viability of human brain pericyte cells (Wilhelmus et al., 2007; Rensink et al., 2002). This is attributed to the uptake of Aβ by pericytes, which inhibits proliferation and causes mitochondrial damage and mitophagy in the pericytes (Kolinko et al., 2021). However, the exact molecular mechanisms underlying the negative effects of Aβ deposition on pericyte numbers remain unclear. Further research is needed to understand how Aβ influences pericyte-mediated capillary contractility and BBB protection. Targeting the PDGFRβ gene may prevent pericyte loss in AD.

#### 3.1.2 Vascular smooth muscle cells

In the brain, smooth muscle cells cover arterioles and exhibit a circumferential band-like morphology (Mazzoni et al., 2015). Recently, its physiological function was revealed to regulate vessel diameter and CBF (Hill et al., 2015), as well as its role as drivers of intramural periarterial drainage (Aldea et al., 2019).

Smooth muscle cells are believed as an important securer for catabolizing Aβ. Previous research has reported that they can internalize Aβ toward lysosomes for clearance (Prior et al., 2000). The low-density lipoprotein receptor-related protein 1 (LRP1), a crucial receptor for Aβ clearance, abundantly exists in vascular smooth muscle cells (Deane et al., 2004; Ruzali et al., 2013). (Kanekiyo et al. 2012) demonstrated that suppression of LRP1 significantly reduced the uptake and degradation of Aβ in human brain vascular smooth muscle cells. Furthermore, deletion of LRP1 in vascular smooth muscle cells accelerated brain Aβ deposition in the APP/PS1 mice at 3 months of age. However, superfluous Aβ accumulation still harms the survival of cerebral smooth muscle cells. The work by (Anzovino et al. 2023) found that both Aβ_1 − 42_ and AβQ22 induced a substantial activation of caspase 8, caspase 9, caspase 3, and caspase 7 for cellular apoptotic signalings via initiating the death receptors (DRs)-DR4 and DR5 and engaging the mitochondrial CytC release. The above studies underscore the potential of cerebrovascular smooth muscle cells as valuable targets for drug interventions in preventing and treating AD. Nonetheless, the precise role and molecular regulatory mechanisms of these central cells in AD pathology remain incompletely understood. Multi-directional research is warranted to elucidate the biological relationship between smooth muscle cells and Aβ metabolism.

### 3.2 Endothelial cell dysfunction

The origin of endothelial cells can be traced back to the multipotent progenitors of embryonic and extraembryonic mesoderm (Marziano et al., 2021), which are among the first vascular cells to differentiate during development (Marziano et al., 2021; Korn and Augustin, 2015). It constitutes the innermost layer of cerebral blood vessels like veins, arteries, and capillaries (Trimm and Red-Horse, 2023), and is directly in contact with the components and cells of blood (Krüger-Genge et al., 2019). Endothelial cells control vessel growth and vascular tone, regulate the regional blood flow, and play a key role in the development and maintenance of the functional circulatory system (Trimm and Red-Horse, 2023; Krüger-Genge et al., 2019; Rucker et al., 2000). Specifically in the brain, endothelial cells coordinate blood supply for the delivery of O_2_ and nutrients and clear metabolic waste for the demand for brain function (Matsuoka et al., 2022; Wei et al., 2023).

AD alters endothelial gene expression (522 upregulated, 501 downregulated) in cortex (Bryant et al., 2023), indicating pathological susceptibility. More importantly, AD induces the endothelial cell subpopulation to exert angiogenesis and immune response in the prefrontal cortex (Lau et al., 2020). In vascular dementia, a subcluster of endothelial cells expresses genes associated with programmed cell death, protein folding response angiogenesis, axonal sprouting, and oligodendrocyte progenitor cell maturation in white matter (Mitroi et al., 2022), suggesting endothelial heterogeneity could inform AD therapeutics. In 3 × -Tg AD mouse model, chronic hypoxic brain condition induces the upregulation of HIF-1α in the endothelial cells (Jung et al., 2023; Grammas et al., 2011). Subsequently, the HIF-1α stimulates the NLR family pyrin domain containing 1 (NLRP1) activation for provoking the adaptor molecule apoptosis-associated speck-like protein containing a CARD (ASC)-caspase 1-IL-1β inflammatory cascade to destroy the vascular function (Jung et al., 2023). Activated HIF-1α also increases the expression of angiopoietin-2 (Ang2), matrix metalloproteinase 2 (MMP2), and caspase 3 but decreases the expression of B-cell lymphoma-extra large (Bcl-xL) for angiogenesis, inflammation, and apoptosis (Grammas et al., 2011). While hypoxia may initially stimulate compensatory angiogenesis, chronic Aβ accumulation causes vascular regression.

*In vitro*, Aβ_1 − 40_ is indicative of endocytotic uptake by brain microvascular endothelial cells and prone to accumulate in acidic cell organelles, such as endosomes and lysosomes (Kandimalla et al., 2009), suggesting that internalized Aβ proteins may influence the cellular physiological function and signal transduction. Furthermore, Aβ_1 − 40_ and Aβ_1 − 42_ inhibited the formation of capillaries of human brain and stimulated capillary degeneration at high doses (Paris et al., 2004b). Aβ_1 − 40_ or the L34V accumulation has been reported to directly induce the cytochrome C (CytC) release from mitochondria to trigger the endothelial cell apoptosis (Fossati et al., 2010). Aβ_1 − 40_ and Aβ_1 − 42_ exposure also trigger the endoplasmic reticulum stress (ERS) response (Fonseca et al., 2014; Chen et al., 2018) to initiate the apoptotic program by activating caspase 12 for apoptosis-inducing factor (AIF) and caspase 3 release (Fonseca et al., 2013). Aβ peptides (e.g., Aβ_1 − 40_, Aβ_25 − 35_, or AβQ22) aggregation at the endothelial cells activate caspase 8 to provoke the release of CytC and AIF, culminating in the activation of caspase 9 and effectors of caspase 3 and caspase 7 (Fossati et al., 2012; Xu et al., 2001). These studies suggest that Aβ detrimentally affects the fate of brain endothelial cells through both exogenous and endogenous apoptotic pathways.

**Figure 2:**
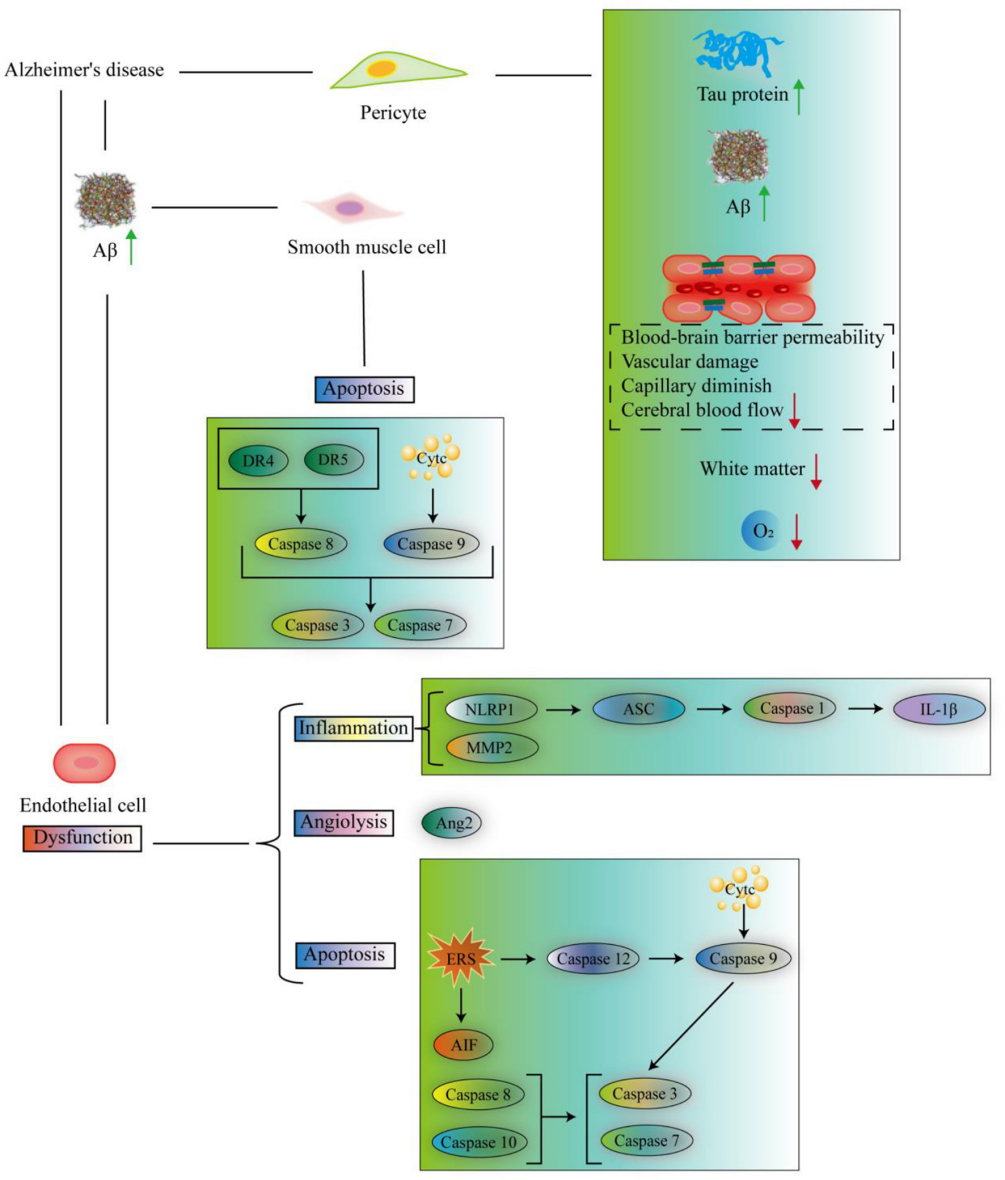
The interrelationship between AD and the changes of various vascular-related cells. Aβ, β-amyloid; DR4, death receptor 4; DR5, death receptor 5; Cytc, cytochrome C; NLRP1, NLR family pyrin domain containing 1; ASC, apoptosis-associated speck-like protein containing a CARD; IL-1β, interleukin-1β; Ang2, angiopoietin-2; ERS, endoplasmic reticulum stress; AIF, apoptosis-inducing factor.

The endothelial mitochondrial content reaches 8%−11% of capillary cytoplasmic volume in the cerebrum (Oldendorf et al., 1977; Parodi-Rullán et al., 2019). As previous biopsy evidence showed that the ratio of mitochondria to endothelium reduced to 2.88%−7.04% in AD (Claudio, 1996). Furthermore, the mitochondrial number and density in the endothelial cells of capillaries are always observed reduced in individuals with AD of the brain regions of the hippocampus, visual cortex, acoustic cortex, and parietal cortices along with the extensive morphological alterations, such as enlarged mitochondria and disruption of the mitochondrial cristae (Baloyannis and Baloyannis, 2012). Subsequently, Aβ_1 − 40_ exacerbates the response of mitochondria-stimulated endoplasmic reticulum (ER) Ca^2+^ release, increases cytC release, promotes ROS accumulation, causes mitochondrial DNA damage, and decreases mitochondrial membrane potential. Thereby, the pathological changes disrupt the redox homeostasis and occur oxidative stress damage in the brain endothelial cells (Xu et al., 2001; Fonseca et al., 2015; Solito et al., 2013). It is evident that AD disrupts the energy demands of endothelial cells, leading to impaired vascular function. Throughout the progression of AD pathology, apoptosis and mitochondrial damage significantly impair endothelial health due to Aβ deposition. Consequently, substantial capillaries with collapsed or degenerated endothelium are observed in the brains of individuals with AD (Kalaria and Hedera, 1995). Focal necrosis of endothelial cells is further indication of BBB leakage (Claudio, 1996). A compromised BBB is a critical early part of AD pathologic events that eventually contribute to cognitive impairment and dementia (van de Haar et al., 2016). Targeting both anti-apoptotic and mitochondrial improvement pathways in cognitive-related regions like the cortex and hippocampus may hold promise in mitigating or halting the development of AD (Figure 2).

## 4 Vascular factors changes

### 4.1 Vasoconstriction

Aβ-generated ROS stimulates endothelin-1 (ET-1) release, activating pericyte ET1 receptor A (ETRA) to constrict capillaries and reduce CBF (Nortley et al., 2019). Hippocampal ET1 injection accelerates Aβ deposition in APP/PS1 mice, while ETRA inhibition prevents vasoconstriction and memory deficits (Wang et al., 2021). Interestingly, ET1 is conversely observed to reduce in the white matter of AD patients, suggesting a potential protective physiological response to decreased white matter perfusion (Barker et al., 2014; Thomas et al., 2015).

### 4.2 Vasodilatation

Nitric oxide (NO) is a gaseous molecule with a wide range of biological activities, synthesized from arginine by nitric oxide synthase (NOS; Robbins and Grisham, 1997). Within the central nervous system, NO produced by vascular endothelium acts as a vasodilator, playing a crucial role in regulating blood pressure, enhancing local blood flow, and reducing vascular resistance in cerebral circulation (Moncada, 1994; Moncada et al., 1991; Katusic and Austin, 2016). Besides, it mediates neuronal survival, synaptic plasticity, vascular smooth muscle relaxation, and endothelial cell permeability (Toda et al., 2009b; Katusic and Austin, 2014). The cerebrovascular NOS-III expression and NO production are decreased along with the characteristics of thickening and hyalinization of the media of small and medium-sized vessels, varying degrees of Aβ deposition, and increased apoptosis of vascular smooth muscle and endothelial cells. This cascade of events may lead to the impairment of vasodilation responses and capillary permeability (De la Monte et al., 2000). In Tg2576 mice, Aβ peptides cripple the biological activity of endothelial nitric oxide synthase (eNOS) by uncoupling the response and thus reduce the NO levels in the cerebral vasculature (Santhanam et al., 2015). Likewise, Inhibition of the eNOS accelerates the generation of Aβ, APP, and β-site APP-cleaving enzyme1 (BACE1) and impairs spatial learning and memory, which is proven in rodent animals and human brain microvascular endothelial cells (Austin et al., 2010, 2013; Austin and Katusic, 2020). Of interest, the NO levels only reduce in the microvessels of eNOS-specific deficiency rather than the brain knockout models (Austin et al., 2010). On the contrary, the content of NO detected from the fresh brain slices in APP/PS1 mice is significantly higher than that of wild-type mice, particularly in the regions around cells containing larger Aβ deposits (Ding et al., 2024). These discrepancies in NO levels can be attributed to variations in detection methods, the heterogeneity of brain tissue subregions and cell types, genetic animal models, and compensatory signal regulation. Nevertheless, NO generation is indeed decreased in the vasculature during the progression of AD.

### 4.3 Angiogenesis

Aberrant angiogenesis may have an amyloidogenic effect in the brain due to compromised BBB clearance of Aβ (Zlokovic, 2008). A clinic study demonstrated that VEGF was markedly increased in the brain regions of white matter, prefrontal lobe, and hippocampus in AD individuals (Barker et al., 2014; Thomas et al., 2015; Kalaria et al., 1998; Tayler et al., 2021). The accumulation of Aβ and hypoperfusion are believed to be key factors leading to the upregulation of VEGF (Thomas et al., 2015; Zhang et al., 2023), suggesting a potential compensatory mechanism to address inadequate vascularity or reduced perfusion in the AD brain. However, despite increased levels of pro-angiogenic factors, there are reports of microvessel degeneration in the cerebral vasculature of AD patients (Fischer et al., 1990; Buée et al., 1994; Paris et al., 2004a; Buée et al., 1997). Notably, VEGF possesses a high affinity for Aβ and is heavily deposited in plaques (Yang et al., 2004), resulting in limited availability of VEGF and impairing angiogenesis (Paris et al., 2004b). Furthermore, trapping Aβ (Bouvet et al., 2023) or exogenous application of VEGF (Martin et al., 2021) is capable of restoring neural synaptic damage by activating the vascular endothelial growth factor receptor 2 (VEGFR2). These interventions highlight the potential therapeutic value of targeting the VEGF pathway to mitigate the detrimental effects of Aβ deposition and promote neural function recovery in AD.

## 5 Angiogenic and anti-angiogenic signalings

### 5.1 Angiogenesis

**Figure 3:**
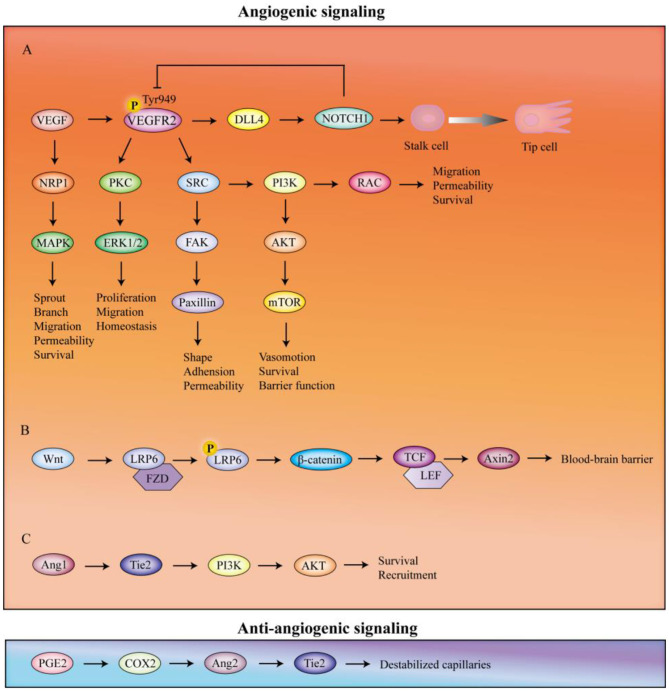
The vascular-related signaling pathways. The angiogenic signalings include VEGF, Wnt, and Ang1-mediated downstreamed molecule. The anti-angiogenic signaling is oriented by PGF2. VEGF, vascular endothelial growth factor; VEGFR2, vascular endothelial growth factor receptor 2; DLL4, Delta-like protein 4; NRP1, neuropilin 1; MAPK, p38 mitogen-activated protein kinases; PKC, protein kinase C; ERK1/2, extracellular-signal-regulated kinases 1 and 2; FAK, focal adhesion kinase; PI3K, phosphoinositide 3-kinase; AKT, protein kinase B; mTOR, mechanistic target of rapamycin; LRP6, low-density lipoprotein receptor-related protein 6; FZD, frizzled; TCF, T-cell factor; LEF, lymphoid enhancer-binding factor; Ang1, angiopoietin-1; PGE2, prostaglandin E2; COX2, cyclooxygenase-2; Ang2, angiopoietin-2.

Angiogenesis is the process of growing new blood vessels from existing vascular systems, involving the proliferation and migration of vascular endothelial cells (Tregub et al., 2022). It must be complemented by remodeling and maturation events, including the removal of redundant vessel segments and cells to shape the new vasculature into an efficient, hierarchical network (Watson et al., 2017), which is a critical process for embryonic and postnatal development, tissue repair, and reproductive function (Parkes et al., 2018). As mentioned, angiogenesis signalings are partially blocked in the AD brain, leading to aggravated vascular degeneration (Tsartsalis et al., 2024). Injection of angiogenesis inhibitor (SU5416) hampers the acquisitive behavior, suggesting angiogenesis is an important component of cognition (Kerr et al., 2010). Based on this evidence, promoting normal neoangiogenesis and improving vascular function may be a pivotal point to restore microvascular circulation in AD (Ambrose, 2015). In this context, we will explore three key angiogenic signalings to provide insights into cerebral angiogenesis and its connection to AD (Figure 3).

#### 5.1.1 VEGF/VEGFR2 signaling pathway

VEGF, now referred to as VEGFA (Dai and Rabie, 2007), promotes angiogenesis, endothelial mitogenesis, migration, and permeability (Melincovici et al., 2018). It is secreted by pericytes and endothelial cells (Procter et al., 2021; Pérez-Gutiérrez and Ferrara, 2023). Numerous factors are involved in VEGFA gene expression, such as peroxisome proliferator-activated receptor-γ coactivator 1α (PGC1α; Arany et al., 2008), oxidative stress (Takahashi et al., 2013), kirsten rat sarcoma viral oncogene homolog (KRAS; Mizukami et al., 2004), and HIF-1 (Forsythe et al., 1996; Georgieva et al., 2023). Hypoxia is the major trigger for its release (Ferrara, 2004; Shweiki et al., 1992). AD-associated hypoxia induces the endothelial cells to up-regulate the activation of HIF-1α (Jung et al., 2023; Grammas et al., 2011), which acts as a detector of the balance between oxygen delivery and energy demand at the level of the cell redox state (LaManna, 2018). Then it binds to the highly conserved hypoxia response element (HRE) of VEGF to enhance the transcription and control the production of vascular endothelial growth factor (Hatakeyama et al., 2020).

VEGF has two main signaling receptors, VEGFR1 and VEGFR2, primarily expressed on endothelial cells. VEGFR2 possesses a higher tyrosine kinase activity than VEGFR-1 and is the main signaling receptor of VEGF, whose activation promotes vascular endothelial cell mitogenesis, migration, and permeability (Melincovici et al., 2018; Apte et al., 2019). Activated VEGF-R2 at the phosphorylated site Tyr949 largely stimulates phospholipase C isoform-γ (PLCγ)-protein kinase C (PKC) pathway for initiating the extracellular-signal-regulated kinases 1 and 2 (ERK1/2) to control proliferation, migration, and homeostasis (Pérez-Gutiérrez and Ferrara, 2023; Takahashi et al., 1999; Simons et al., 2016). VEGFR2 also activates SRC to induce the phosphoinositide 3-kinase (PI3K) and focal adhesion kinase (FAK) for signal cascade (Simons et al., 2016). Among these, the PI3K downstream molecule protein kinase B (PKB, also known as AKT)-mechanistic target of rapamycin (mTOR) pathway is crucial for cell survival, vasomotion, and barrier function (Zhuang et al., 2013). Small GTP-binding protein RAC exerts the function of survival, vascular permeability, and cellular migration (Tan et al., 2008). Besides, the paxillin, the substrate of the FAK, orchestrates the shape and adhesion of endothelium (Westhoff et al., 2004) and the vascular permeability (Chen et al., 2012). Delta-like protein 4 (DLL4) is stimulated by VEGFR2 phosphorylation and mediates the activation of NOTCH1 in adjacent stalk cells (specified by endothelial cells to form the nascent sprout body; Pérez-Gutiérrez and Ferrara, 2023), which allows stalk cells to revert to tip cells (a highly motile type of endothelial cells; Benedito et al., 2009) and controls sprouting angiogenesis and artery formation (Pitulescu et al., 2017). Activated NOTCH1, in turn, maintains the expression of VEGFR2 in stalk cells to suppress the tip cell phenotype and establish stable, lumenized vasculature (Simons et al., 2016).

Neuropilin 1 (NRP1) is a transmembrane glycoprotein that is critical for VEGF-induced sprouting and branching of endothelial cells (Gomez et al., 2023; Kawamura et al., 2008). It can bind to VEGFA to activate the p38 mitogen-activated protein kinases (MAPK) for endothelial migration, permeability, and survival (Melincovici et al., 2018; Pérez-Gutiérrez and Ferrara, 2023; Kawamura et al., 2008).

#### 5.1.2 Wnt/β-catenin signaling pathway

The canonical Wnt signaling pathway drives cerebrum-specific angiogenesis and shapes vascular development (Daneman et al., 2009; Gupta et al., 2021), which is crucial for angiogenesis of the central nervous system and BBB formation (Hübner et al., 2018). Defects of this signaling pathway could lead to the reduction of the vascular number, loss of tiny capillaries, and formation of hemorrhagic vascular malformation (Korn and Augustin, 2015). Wnt can bind to the receptor Frizzled (Fzd) and co-receptor low-density lipoprotein receptor-related protein 6 (LRP6) complex to phosphorylate LRP6. Then the stabilized β-catenin engages with T-cell factor (TCF)/lymphoid enhancer-binding factor (LEF) transcription factors (Schuijers et al., 2014) to activate targeted gene Axin2 transcription (Lustig et al., 2002). Activating this signaling can mitigate and repair the Aβ-induced BBB malfunction in the APP/PS1 mouse model (Wang et al., 2022). Therefore, targeting the Wnt/β-catenin pathway could represent a promising molecular approach to address vascular dysfunction in AD.

#### 5.1.3 Ang1/Tie signaling pathway

The Ang ligands and Tie receptors constitute the second vascular tissue-specific receptor tyrosine kinase system, which is known to be indispensable for embryonic vessel formation and maturation as well as the regulation of adult vascular homeostasis (Augustin et al., 2009). Consequently, this signaling pathway involving the Tie receptors and their angiopoietin ligands acts as a context-dependent regulator of vascular remodeling (Eklund and Olsen, 2006). In normal adult tissues, Ang1, predominantly expressed in capillary pericytes (Sundberg et al., 2002; Wakui et al., 2006), has an antiapoptotic effect on endothelial cell survival and maintains the integrity through inhibiting the Ang2 expression (Ucuzian et al., 2010). Activation of the Tie2 receptor by Ang1 triggers occludin expression (Hori et al., 2004), crucial for preserving capillary mechanical stability. The primary downstream effector of Tie2 is PI3K, which facilitates AKT-mediated endothelial cell survival and recruitment of mural cells (Augustin et al., 2009).

### 5.2 Angiolysis

Capillary density is determined by the dynamic equilibrium of angiogenesis and angiolysis (LaManna, 2018). The physiological regression of cerebral vessels is predominantly mediated by the failure of endothelial cells to relocate following vascular blockage, ultimately leading to their apoptosis (Tregub et al., 2022; Korn and Augustin, 2015). Pruning of immature vessels is an important mechanism of antiangiogenic vessel-normalizing therapies.

Ang2, primarily produced by endothelial cells and pericytes (Dore-Duffy and LaManna, 2007), may also have an anti-angiogenic effect as an antagonist of constitutive Ang1/Tie2 signaling in the absence of VEGF (LaManna, 2018; Augustin et al., 2009). It is transiently elevated in endothelial cells and pericytes under hypoxia (Pichiule and LaManna, 2002) due to increased expression of cyclooxygenase-2 (COX-2) and consequently elevated levels of prostaglandin E2 (PGE2; Pichiule et al., 2004). The activated Ang2 binds to the Tie2 receptor to prevent Ang1 activation and destabilize the capillaries (Dore-Duffy and LaManna, 2007). This can lead to endothelial cell apoptosis, the formation of disorganized and immature new blood vessels, as well as vessel regression (Huang and Bao, 2004).

## 6 The exercise effect on cerebrovascular function improvement in AD

Exercise has emerged as a promising non-pharmacological intervention for preventing and improving several clinical symptoms of AD with minimal risk or adverse effects (2023; Laurin et al., 2001; De Miguel et al., 2021; Intlekofer and Cotman, 2013; Alzheimer's disease facts and figures, 2023).

### 6.1 Exercise promotes cerebral angiogenesis

Exercise is a powerful driver of physiological angiogenesis, which substantially increases the cerebrovascular number (Isaacs et al., 1992; Huang et al., 2021; Jordão et al., 2021; Padilla et al., 2011). In skeletal muscle, exercise expands vasculature via ATF3/4^+^ proliferation (Fan et al., 2021). This implies that exercise is capable of enhancing the vascularization potential of endothelial cells, which may also work on the cerebrum tissue. Three day exercise can enhance the VEGF expression in the cortex and hippocampus and endothelial cell proliferation in middle-aged female mice, which promotes beneficial structural modifications in the cerebrovascular system (Latimer et al., 2011). Long-term training enhances the expression of platelet-derived growth factor subunit A (PDGFA), PDGFB, VEGF, Ang1, and Ang2 in the cortex of ovariectomized rats (Yoon et al., 2023). Moreover, exercise prevents stroke-induced microvessel density reduction in the striatum and cortex by activating of Tie-2 and AKT, thereby ameliorating neurologic deficits and infarct volume in the frontoparietal cortex and dorsolateral striatum (Ding et al., 2004; Zhang et al., 2013). Exercise also boosts the number of capillaries in the cortex and improves the cerebral blood supply in hypertension (Jordão et al., 2021) and aged rats (Wang et al., 2015; Chen et al., 2020), potentially via neurotransmitters and their signaling pathways.

L-lactate, as a byproduct of exercise, exerts various neurobiological effects (Brooks et al., 2022a,b). In AD rodent models, the level of L-lactate is always observed to decline in the hippocampal and cortical regions (Lu W. T. et al., 2019; Lu et al., 2015; Zhang M. et al., 2018). The brain L-lactate may not just be the pathological diagnostic biomarker for AD but also plays a pivotal role in this disease, although the exact mechanism is still unclear. An exogenous supplement of L-lactate is reported to enhance the vascular density in the dentate gyrus of the hippocampus via the secretion of VEGF (Morland et al., 2017). Exercise can elevate the brain L-lactate content through monocarboxylate transporter (MCT1; Pierre and Pellerin, 2005; Steinman et al., 2016; Aveseh et al., 2014) and astrocytic glycolysis (Magistretti and Allaman, 2015; Newman et al., 2011; Suzuki et al., 2011). Morland et al. reported that exercise activates the hippocampal L-lactate receptor, Gi-protein-coupled receptor 81 (GPR81, also known as HCAR1; Ahmed et al., 2009; Morland et al., 2015; Meng et al., 2024), phosphorylating the ERK1/2 to up-regulate VEGF and microvascular density via L-lactate (Morland et al., 2017).

**Figure 4:**
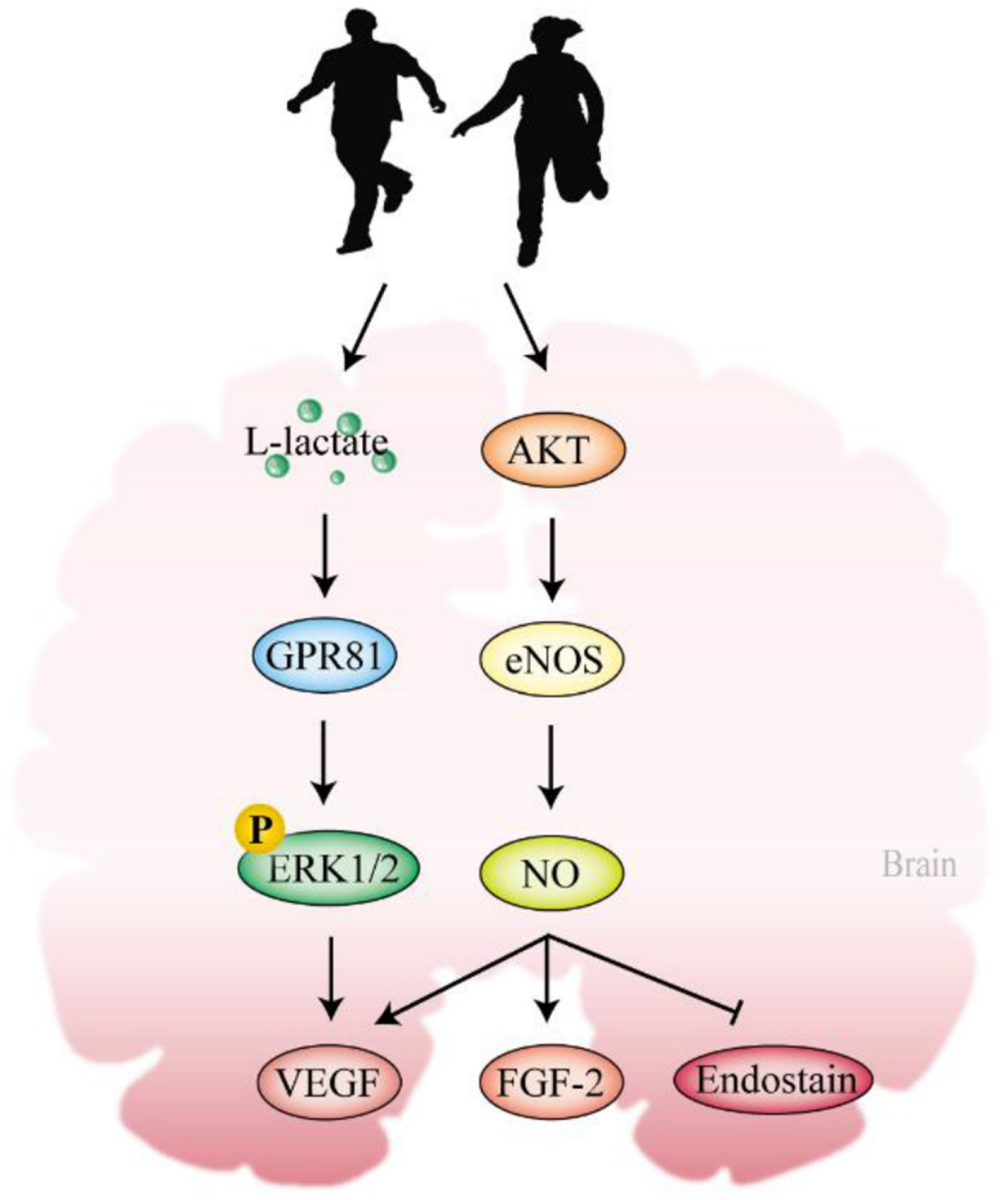
Exercise regulates cerebrovascular angiogenesis. Exercise mainly produces L-lactate and activated AKT to provoke the angiogenic signalings in the brain. AKT, protein kinase B; GPR81, Gi-protein-coupled receptor 81; ERK1/2, extracellular-signal-regulated kinases 1 and 2; VEGF, vascular endothelial growth factor; eNOS, endothelial nitric oxide synthase; NO, nitric oxide; FGF-2, fibroblast growth factor-2.

Exercise enhances the activity of eNOS to synthesize endogenous NO via AKT action (Bolduc et al., 2013). This increase in NO production stimulates the release of VEGF and fibroblast growth factor-2 (FGF-2), inhibits endostatin production, and results in greater total length, volume, and surface area of cortical capillaries, ultimately improving cognitive function (Viboolvorakul and Patumraj, 2014; Zang et al., 2023; Figure 4). This body of evidence supports the notion that exercise enhances cerebral perfusion and metabolism, promotes cerebrovascular adaptation, and has the potential to reshape cerebral vascular function in the context of AD.

Despite these promising findings, key questions regarding exercise effect in AD remain: Does exercise truly benefit the vascular network in the AD brain? Can exercise promote neovascularization in AD through explicit L-lactate and NO-dependent signaling pathways? Abnormal microvessel formation and regression may play a role in AD pathogenesis (Li S. et al., 2021; Tregub et al., 2022). How does exercise prune useless string vessels and remodel the vascular function to ensure cerebral plasticity in AD? Moreover, what role do L-lactate and NO play when exercise interacts with classical angiogenic and anti-angiogenic signaling pathways during AD? Further research is needed to resolve these.

### 6.2 Exercise regulates CBF

During exercise, CBF and cerebrovascular reactivity increase in response to elevated CO_2_ (Olver et al., 2015). The CBF velocity in the middle cerebral artery linearly rises with exercise intensity, peaking at about 60%−70% of maximum aerobic capacity, then quickly returns to baseline levels post-exercise and remains stable thereafter (Nishijima et al., 2016). Moreover, cerebral vascular response performance returns to normal levels within half an hour of exercise cessation (Steventon et al., 2018), indicating that exercise can regulate cerebrovascular reactivity. Compared to sedentary individuals, exercise participants exhibit a 17% higher middle cerebral artery blood flow velocity (Ainslie et al., 2008). A cross-sectional study has revealed that Tai Chi recovers the carotid artery hemodynamics in the old adults for a more adequate blood supply to the brain (Li L. et al., 2021). A 12-week rhythmic physical task (multitask movement music therapy) can increase the CBF of prefrontal cortex in the MCI patients for the cognitive performance improvement (Shimizu et al., 2018). Exercise improves CBF through multiple mechanisms. From the repair of vascular structure, exercise has been shown to normalize hemodynamic fluctuations, reduce abnormal vascular density, and modulate capillary hemodynamics in AD patients, as evidenced by neuroimaging techniques (Lu et al., 2019). At the molecular signaling levels, long-term regular exercise excites the eNOS to transform vasoconstriction into vasodilation to mitigate cerebrovascular dysfunction in APP/PS1 mice (Hong et al., 2020). Additionally, exercise, especially the moderate-intensity pattern (Zeng et al., 2023), can produce a higher level of the blood flow shear force, which activates the AKT to phosphorylate the eNOS for increasing the bioavailability of NO (Wu et al., 2019) and inhibits the secretion of ET-1 at the same time (Ziegler et al., 1998). From the metabolic perspective, the blocked brain capillaries caused by interruption of vascular flow are associated with the formation of hypoxic pockets in AD. Exercise can reduce the approximately 52% hypoxic pockets to improve the local supply of oxygen and thereby the restoration of blood flow in the cerebral cortex (M. Beinlich et al., 2024).

However, the exercise diversity, including patterns, intensity, duration, environment, and individual qualities, leads to differences in the effects of exercise on CBF regulation (Huang et al., 2022; Frederiksen et al., 2018). For instance, a pilot study has reported that a 16-week aerobic exercise (AE) regimen (cross trainer, bicycle, and treadmill) is insufficient for CBF in AD individuals (van der Kleij et al., 2018). A 3-month wheel running also has no effect on the baseline CBF in APP/PS1 mice (Falkenhain et al., 2020). Exercise intensity is the primary factor affecting the arterial hemodynamic signalings, such as shear stress, blood pressure, and circumferential stretch strain (Chien, 2007; Zhou et al., 2014). Arterial hemodynamic signalings control the vascular endothelial repair processes, which affect the NO production for vasodilation (Liu et al., 2018; Wang et al., 2019). Thus, despite inconsistent outcomes in AD models, optimizing intensity is critical. moderate-intensity exercise may be optimal due to its ability to enhance hemodynamic signaling.

## 7 The effect of exercise on the cerebrovascular function related cells

### 7.1 Perithelial cells

Recent animal research has suggested that 16-week treadmill exercise can increase approximately 60% PDGFRβ and 95% neural/glial antigen 2 (NG2) by stimulating the neuron to release miR-532-5p for the downregulation of erythropoietin-producing hepatocellular carcinoma A4 (EPHA4). MTT assay analysis further proves the cell proliferation in pericytes. Concurrently, enhanced pericyte low-density lipoprotein receptor-related protein 1 (LRP1) expression facilitates Aβ clearance across the blood-brain barrier, synergistically alleviating neurovascular dysfunction. Exercise-induced exosomal miR-532-5p rescues pericyte loss and enhances BBB functional integrity (Liang et al., 2023).

### 7.2 Vascular smooth muscle cells

The effects of exercise on cerebral smooth muscle cells are primarily observed in hypertensive animal models. (Shi et al. 2016) demonstrated that a 12-week treadmill exercise regimen efficiently prevents the enhancement of coupling of ryanodine receptors-large-conductance Ca^2+^-activated K^+^ channel (BK_Ca_) and inhibits the release of Ca^2+^ from ryanodine receptors in the smooth muscle cells to restore arterial function. This pattern of AE also effectively relieves the tension in cerebral arteries by inhibiting Ang2-AT_1_ receptor (AT_1_R)-a-kinase anchoring protein 150 (AKAP150)-protein kinase Cα (PKCα) signaling (Zhang et al., 2024). These findings position exercise as a non-pharmacological strategy to reset maladaptive cerebrovascular signaling pathways in hypertension, offering dual benefits of improved arterial diastolic function and attenuated structural remodeling.

### 7.3 Endothelial cells

**Figure 5:**
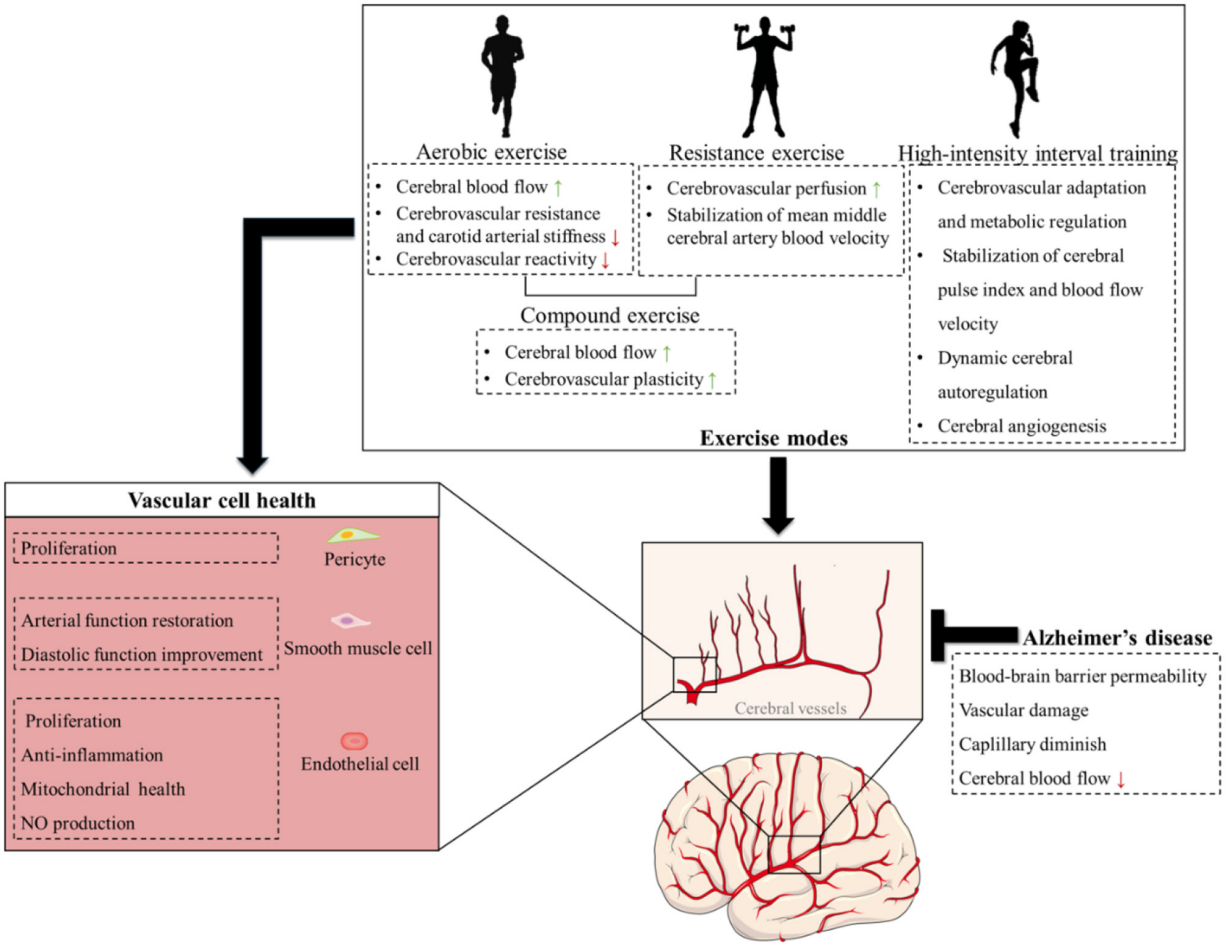
Cerebrovascular mechanisms of different exercise modes in Alzheimer's disease. This schematic illustrates that Alzheimer's disease triggers cerebrovascular dysfunction, characterized by damage to the cerebrovascular microstructure, compromised blood-brain barrier integrity, and reduced cerebral blood flow. Different exercise modalities (aerobic exercise, resistance exercise, high-intensity interval training, and compound training) enhance cerebrovascular health by optimizing cerebrovascular structure and function to mitigate Alzheimer's disease pathology. At the cell levels, these exercises target the metabolism of endothelial cell, smooth muscle cell, and pericyte to promote vascular health.

The impact of exercise on brain microvascular endothelial cells appears genotype-dependent. It reduces the endothelial expression of sirtuin 1 (SIRT1) in APOE-ε4, while increasing SIRT1 levels in APOE-ε3 carriers, which potentially weakens inherent barrier dysfunction and glucose hypometabolism associated with AD risk (Weber et al., 2025). Additionally, exercise has a beneficial effect on the cerebral endothelial cells health. For example, exercise can improve the proliferation of cerebral endothelial cells to enrich the cerebral vascular branches for vascular plasticity (Latimer et al., 2011). Furthermore, exercise-induced plasma components, particularly elevated clusterin, mediate anti-inflammatory effects on cerebral endothelium by suppressing inflammatory signaling in vascular endothelial cells, while simultaneously promoting neurogenesis and cognitive enhancement (De Miguel et al., 2021). Besides anti-inflammation, exercise improves mitochondrial metabolism by inhibiting mitochondrial fission, thus restoring endothelial-dependent vasodilation in the mesenteric artery endothelium of hypertensive rodent models (Li G. et al., 2022). Exercise also up-regulates adenosine monophosphate-activated protein kinase (AMPK), leading to the activation of downstream eNOS cascades for the release of endothelial protective product-NO. Interestingly, the effect of AE on the phosphorylation of AMPK particularly depends on the intensity increase, whereas the effect of resistance exercise (RE) depends on both intensity and duration (Liu et al., 2023). For instance, higher intensity levels yield greater stimulation of endothelial progenitor cells at intensities ranging from 60% to 80% of maximum, potentially resulting in increased NO production (Ribeiro et al., 2017). These findings collectively highlight that exercise may serve as a potent modulator of endothelial homeostasis through genetic interaction, anti-inflammation, mitochondrial regulation, and systemic protein-mediated crosstalk. Notably, the exercise type has a distinct influence on the endothelial cells. Different exercise frequencies reveal that AE and RE exert the most significant effects on circulating endothelial progenitor cells in acute trials (Ferentinos et al., 2022a). High-intensity interval training (HIIT) appears particularly beneficial for aging populations, individuals with chronic heart failure, and hypertensive patients, mobilizing endothelial progenitor cells to improve endothelial function and reduce arterial stiffness in chronic interventions, followed by moderate-intensity AE (Ferentinos et al., 2022a,b). These evidences underscore the need for personalized exercise regimens and further research to explore whether exercise exerts multifaceted beneficial effects on cerebral endothelial cells through diverse molecular mechanisms in AD, as well as to discuss the impact of different exercise patterns on endothelial cell mobilization (Figure 5).

## 8 Exercise pattern for treating AD cerebrovascular dysfunction

### 8.1 Aerobic exercise (AE)

AE, also known as endurance activity or cardiovascular exercise, is defined by the American College of Sports Medicine (ACSM) as any activity that uses large muscle groups is maintainable continuously, and has a rhythmic nature (Wahid et al., 2016). In the older and MCI adults, AE, such as moderate-intensity walking, enhances the functional connectivity within the hippocampus, posterior cortex, and cingulate cortex (Burdette et al., 2010; Chirles et al., 2017), implying its role in improving the integration of memory consolidation and default mode network regulation. Additionally, AE enhances the CBF in the hippocampal region of hypertensive APOE4 carriers, reducing the genetic risk for AD (Kaufman et al., 2021). Exercise like bike and treadmill increases resting CBF in the anterior cingulate cortex and hippocampus, facilitating neuroplasticity and thereby improving the immediate and delayed memory in middle-aged and elderly adults (Chapman et al., 2013). Furthermore, in older individuals, a longitudinal cycling intervention is reported to reduce the cerebral pulsatility index by normalizing vascular compliance to pulsatile flow following exercise (Akazawa et al., 2018). Submaximal AE (40% VO_2max_ or 65 W) increases the CBF and cerebrovascular conductance (calculated as CBF/cerebral perfusion pressure) while reducing CVR, thereby conferring cerebrovascular benefits (Lake et al., 2022). Critically, higher-intensity AE (>60% VO_2_max__) reduces the carotid arterial stiffness, CVR, and central arterial stiffness, linking cerebrovascular improvement to executive function (Ainslie et al., 2008; Tomoto et al., 2023b; Kleinloog et al., 2019) as well as memory enhancement (Tomoto and Zhang, 2024). In the amnestic MCI participants, AE, such as brisk walking, enhances cerebrovascular reactivity by augmenting the cerebral vasomotor response to hypercapnia (Tomoto et al., 2021b). AE (>75% HR_max_) increases the CBF and decreases the pulsatility index, thereby improving the cerebral perfusion for the cognitive performance (Tomoto et al., 2021a). Exercise (e.g., brisk uphill walking) also redistributes CBF by enhancing anterior cingulate perfusion while reducing posterior cingulate perfusion, which is associated with improved memory (Thomas et al., 2020). This underscores the necessity for personalized AE prescriptions in AD populations with compromised cerebrovascular reserve.

### 8.2 Resistance exercise (RE)

RE is a form of exercise that increases muscular strength and endurance by exercising a muscle or muscle group against resistance (e.g., push-ups, squats, crunches, pull-ups, and weightlifting; Sutton, 2022). Unlike AE, RE is characterized by transient and bidirectional physiological extremes to produce a profoundly hemodynamic response. It results in a markedly different hemodynamic response, including phasic perturbations in blood pressure, that affect CBF regulation (Perry and Lucas, 2021). Compared to sedentary peers, elderly individuals who regularly engage in RE, such as weight lifting or calisthenics, demonstrate superior cerebrovascular perfusion and function (Xu et al., 2014). Recent study indicates that habitual RE (e.g., leg extensions at 60% 1 RM) stabilizes mean middle cerebral artery blood velocity, suggesting enhanced CBF regulation (Korad et al., 2024). Interestingly, while young adults exhibit similar cerebrovascular reactivity during both AE and RE, AE demonstrates higher cerebrovascular conductance (Corkery et al., 2021). Despite these findings, the impact of RE on cerebrovascular function in AD patients was still obscure.

### 8.3 High-intensity interval training (HIIT)

HIIT involves repeated short to long bouts of rather high intensity exercise (equal or superior to maximal lactate steady-state velocity) interspersed with recovery periods (light exercise or rest; Billat, 2001). Modalities include jumping jacks, high knees, sprinting combined with walking, bicycle sprinting with slow riding, rapid alternating lateral lunges, burpees, and Tabata. Compared to traditional AE, time-efficient HIIT offers superior benefits in terms of metabolic, cardiac, vascular adaptations, pulmonary function, and aerobic fitness (Lucas et al., 2015; Weston et al., 2022). Rodent study indicates that HIIT ameliorates AD-like pathology (Feng et al., 2023), highlighting its potential role in the prevention and treatment of AD. Acute HIIT has been shown to increase both deoxygenated and oxygenated hemoglobin levels and cerebrovascular reactivity, while reducing blood velocity in the middle cerebral artery, thereby enhancing dynamic cerebral autoregulation (Komiyama et al., 2020; Northey et al., 2019). This suggests that acute HIIT plays a significant role in regulating cerebrovascular responses. Compared to the AE, regular HIIT stabilizes the cerebral pulsatility index, resistivity index, and dynamic cerebral autoregulation, maintaining CBF and velocity post-exercise (Tsukamoto et al., 2019; Whitaker et al., 2022). Besides, HIIT stimulates cerebral microangiogenesis in wild-type mice via L-lactate and its receptor (Morland et al., 2017), which supports the neurogenesis and neuroplasticity (Hu et al., 2021). Generally, multiple lines of evidence suggest that HIIT indeed boosts the cerebral vascular function and benefited cognition. However, there is a paucity of research investigating whether the regulatory effects of HIIT on cerebrovascular metabolism and genesis are applicable to the development of AD. Further studies are needed to explore its relevance to AD-related vascular metabolism.

### 8.4 Compound training (CT)

CT is a type of strength training movement that engages multiple muscle groups and joints simultaneously (e.g., squats, deadlifts, bench press, push-ups and pull-ups). These exercises are fundamental in building overall strength, enhancing muscle mass, and improving functional fitness. Unlike isolation exercises, which target a single muscle group, compound exercises provide a more comprehensive workout (Mihalik et al., 2008). This method usually involves strength training with high resistances (70%−90% 1 RM) in 1 session and ballistic training or plyometric training with lower resistances (e.g., ~30% 1 RM or with body mass) on a different day. A randomized clinical research comparing 9-week AE (walking) and CT (strength+walking) in AD patients found that CT was superior for improving global cognition, visual/verbal memory, and executive function (Bossers et al., 2015). This superiority may stem from CT combining the benefits of both exercise modalities, resulting in improved CBF and cerebrovascular plasticity (Bliss et al., 2021). These findings hint at the need to consider the beneficial physiological effects of various forms of exercise on cerebral blood vessels when formulating exercise prescriptions, leading to more precise exercise strategies tailored to patients' needs (Tables 1, 2; Figure 5).

**Table 1:** Summary of exercise interventions for AD-related cerebrovascular dysfunction.

**Type**	**Subjects type**	**Exercise intervention forms**	**Intervention duration**	**Key outcomes**	**Observed effects**	**Physiological mechanisms**
AE	Cognitively healthy older adults hypertensive APOE4 carriers; Amnestic MCI; Low-active healthy older adults; Sedentary patients with amnestic MCI	Aerobics; Brisk uphill walking; Cycling; Brisk walking	150 min/week × 3–5 day/week × 52 weeks (40%−55% HRR); 25–35 min/session × 3–5 sessions/week × 12 months (75%−85% HRmax); 35–45 min/day × 4–6 day/week × 12 weeks (65%−80% HR_peak_); 30–35 min/session × 3–4 sessions/week × 12 months (75%−85% HR_max_)	HBF; CBF; CAS/CBF/CVRi; CBF/CVCi/MAP	Improved memory and executive function; Improved vascular compliance;	HBF↑ (Kaufman et al., 2021); CBF↑ (Tomoto et al., 2021a); Cerebral pulsatility index↓ (Akazawa et al., 2018); Cerebrovascular reactivity↑ (Tomoto et al., 2021a)
RE	Older adults; Young healthy adult; Young healthy sedentary men	Lifting weights or calisthenics; Unilateral leg extensions; Weightlifting	Lifting weights or calisthenics ≥ once per week; 10 repetitions of unilateral leg extensions at 60% 1 RM to a tempo of 15 bpm, which equates to a repetition cycle length of 4 s (2 s per concentric and eccentric phase); 3 multi-joint and 5 single joint exercises machines and free weight (2 day/week × 12 weeks)	CBF; MCAv/MAP/MCAVmean; β-Stiffness index/CBF/pulsatility index	Hemodynamic adaptation to blood pressure fluctuations; Enhanced vascular response;	Cerebrovascular perfusion↑ (Xu et al., 2014); Stabilized middle cerebral artery blood velocity (Korad et al., 2024); CBF regulation (Nakamura and Muraoka, 2018)
HIIT	Inactive Adults; Female breast cancer survivors; Young healthy adults; Healthy men	Treadmill walking; Cycle ergometer; Recumbent stepper; Cycling	4-min interval at 90%−95% HR_max_ with 3-min active recovery 70% HR_max_; 30-s intervals at ~90% HR_max_ or ~105% peak power with 2-min active recovery; 10 min of 1-min high intensity (~70% estimated maximal Watts) and active recovery (10% estimated maximal Watts) intervals; 4-min bouts of exercise at 80%−90% maximal Watts interspaced by 3-min bouts at 50%−60% maximal Watts × 4 sets	Oxygenated hemoglobin/CBF; MCAv; MCAv/MAP; MAP/MCAv_mean_	Enhanced metabolic efficiency Improved dynamic cerebral autoregulation and cognitive performance; Enhanced vascular response; Cerebral microangiogenesis↑	Increased brain activation and oxygenated hemoglobin (Coetsee and Terblanche, 2017); Cerebrovascular reactivity and CBF↑ (Northey et al., 2019); Stabilized cerebral pulsatility index (Tsukamoto et al., 2019; Whitaker et al., 2022)
CT	Older hospitalized patients; MCI females; Probable MCI females	AE+RE; AE+RE; AE+RE	AE (2 daily sessions of 20 min duration during 5–7 consecutive days, 50% HR_max_)+RE (2–3 sets of 8–10 repetitions with a load equivalent to 30%−60% of the estimated 1 RM); 1 × 60 min/week RE+2 × 60 min/week AE × 12 months; 2 day/week AE (outdoor walking, 40%HRR)+RE (2 sets of 6–8 repetitions) × 6 months	CBF; MCAv/CBF; MCAv_mean_/hippocampal volumes	Improved executive function and cognition; Improved cognition, memory, and executive function; Increased verbal memory and learning	CBF↑ (Sáez de Asteasu et al., 2019); Cerebrovascular plasticity↑ (Nagamatsu et al., 2012); Hippocampal volumes↑ (ten Brinke et al., 2015);

AE, aerobic exercise; RE, resistance exercise; HIIT, high-intensity interval training; CT, compound training; CBF, cerebral blood flow; CVR, cerebrovascular resistance; CVCi, cerebrovascular conductance index, CVCi = CBFV/MAP; CVRi, cerebrovascular resistance index; CVRi = MAP/MCAv; CAS, central arterial stiffness; HR, heart rate; HR_max_, maximal heart rate; HRR, heart rate reserve; HBF, hippocampal blood flow; HIIT, high-intensity interval training; MCAv, middle cerebral artery blood flow velocity; MAP, mean arterial pressure. The symbols "↑" represent an increase and "↓" represent a decrease.

**Table 2:** Glossary of terms.

**Term**	**Definition**	**Common forms**
AE	Also called endurance activity or cardiovascular exercise, any activity that uses large muscle groups is maintainable continuously, and has a rhythmic nature (Wahid et al., 2016)	Walking, jogging, swimming, cycling, aerobics
RE	A form of exercise that increases muscular strength and endurance by exercising a muscle or muscle group against resistance (Sutton, 2022)	Push-ups, squats, crunches, pull-ups, weightlifting
HIIT	Involves repeated short to long bouts of rather high intensity exercise (equal or superior to maximal lactate steady-state velocity) interspersed with recovery periods (light exercise or rest) (Billat, 2001)	Jumping jacks, high knees, sprinting combined with walking, bicycle sprinting with slow riding, rapid alternating lateral lunges, burpees, Tabata
CT	A type of strength training movement that engages multiple muscle groups and joints simultaneously (Mihalik et al., 2008)	Squats, deadlifts, bench press, push-ups, pull-ups

AE, aerobic exercise; RE, resistance exercise; HIIT, high-intensity interval training; CT, compound training.

## 9 Prospect and conclusion

Cerebrovascular dysfunction, characterized by reduced cerebral blood flow, vascular degeneration, abnormal vascular pruning, and altered vascular factors, is a common feature associated with the onset of AD. Clinical observations have indicated that cerebrovascular density may remain unchanged or even show compensatory angiogenesis in individuals with AD, highlighting the complexity and inconsistency of pathological changes in the disease. Furthermore, significant individual variations exist in these vascular alterations.

Sedentary behavior is a well-established risk factor for AD, making exercise interventions a recommended strategy for prevention and symptom management. However, the precise mechanisms underlying the protective effects of exercise on cerebral circulation and neuronal tissue are not fully elucidated. Although exercise known to regulate CBF and promote microangiogenesis, translating these biological effects into effective AD therapeutics requires further mechanistic investigation.

Exercise parameters, such as exercise volume, frequency, duration, type, and intensity, influence brain blood flow and produce distinct cerebrovascular effect (Tomoto and Zhang, 2024; Perry et al., 2020; Smail et al., 2023). This heterogeneity implies that exercise prescriptions cannot be standardized for cerebrovascular outcomes. Therefore, understanding the role of exercise in cerebrovascular function cannot be based solely on a single mode of exercise, even within the same exercise type. In the meantime, large-scale, long-term follow-up studies are necessary to clarify the differentiated effects of exercise on various pathological stages of AD. Such studies will provide significant clinical value for formulating stepwise exercise prescriptions. Further research is warranted to explore the potential benefits of specialized exercise on cerebrovascular function in AD by constructing a multimodal assessment system and uncovering the specific pathways through which tailored exercise programs impact vascular health and neuronal integrity. Understanding these mechanisms and establishing precise medical models for exercise intervention could contribute to the development of targeted therapies aimed at preserving cerebrovascular health and potentially slowing the progression of AD.

In clinical practice, implementing an exercise regimen faces a dual challenge: Firstly, adherence to exercise among patients with AD is adversely affected by primary symptoms, such as cognitive decline and impaired exercise capacity. Therefore, early exercise intervention for individuals in the aging and MCI population may be a more feasible strategy for preventing or delaying the progression of AD pathology. Secondly, the mechanism of synergy between exercise therapy and targeted drug pharmacological methods has not yet been fully clarified. The ability of exercise to enhance the permeability of BBB may have an impact on the efficiency of drug delivery.
